# Active ageing within the nursing home: a study in Flanders, Belgium

**DOI:** 10.1007/s10433-016-0374-3

**Published:** 2016-04-15

**Authors:** Lien Van Malderen, Patricia De Vriendt, Tony Mets, Ellen Gorus

**Affiliations:** 1 Research Foundation Flanders (FWO-Vlaanderen), Brussels, Belgium; 2 Gerontology Department, Frailty in Ageing Research Group, Vrije Universiteit Brussel, Laarbeeklaan 103, 1090 Brussels, Belgium; 3 Arteveldehogeschool, Voetweg 66, 9000 Ghent, Belgium; 4 Geriatric Department, Universitair Ziekenhuis Brussel, Laarbeeklaan 101, 1090 Brussels, Belgium

**Keywords:** Quality of life, Residential care, Satisfaction, Quality of care

## Abstract

Nursing homes should support residents’ quality of life (QoL). It remains vague, however, how these facilities can create a QoL enhancing environment. Active ageing (AA) is a useful framework in this context, since it provides a multidimensional set of determinants that enhance QoL. This study examined the current status of AA in nursing homes in Flanders, Belgium. A sample of 383 randomly recruited residents was surveyed on the subjective importance and experienced reality of the AA determinants as well as on QoL. Based on descriptive analyses, residents appeared to have a positive QoL and a moderately positive appraisal of the extent to which nursing homes provide a multidimensional environment to enhance their QoL. Multivariate analyses showed that residents’ nursing home active ageing (NHAA) experience was positively related to their QoL and explained 20 % of its variance. Specifically, psychological factors and participation related positively to QoL. Demographic variables showed no relationships with QoL, while educational level related negatively to the NHAA experience. Currently, in Flanders, nursing homes are on their way to working according to the AA vision, but further efforts are still needed.

## Introduction

Over the last century, the population of Europe has become the oldest in the world (Walker 2008). Similar to other European countries (Eurostat 2012), 19 % of the population in Flanders—the Dutch- speaking region in Belgium with 6 million inhabitants—are aged 65 years and above (Research Department Flemish Government 2014). Seven percent of this older Flemish population are living in nursing homes. In the next 20 years, the number of people in need of residential care is expected to rise continuously (Huber et al. 2009).

Quality of life (QoL) has become an important outcome to determine the quality of healthcare and service delivery towards older people. QoL is defined by the World Health Organization (WHO 1995, p. 1403) as *“the individual’s perception of his or her position in life in the context of the culture and value systems in which they live and in relation to their goals”*. Gilhooly et al. (2005, p. 18) described it as *“the degree of satisfaction over all areas in life important to the individual concerned”*. These definitions underline the multidimensional nature of QoL, with various life domains influencing the individual’s overall perception and interacting with each other, including, for example, relationships, psychological wellbeing, health, and received care (Bernheim 1999; Bowling et al. 2003; Higgs et al. 2005; Kelley-Gillespie 2009; Marinelli and Plummer 1999; Murphy et al. 2007). QoL is individually defined (Bernheim 1999; Bowling et al. 2003; Bowling and Gabriel 2004), and the importance of each dimension differs from person to person (Bernheim 1999; Bowling and Gabriel 2004). QoL is also dynamic, as the experienced reality is compared with past experiences and expectations for the future. This analysis changes over time, implying that the meaning of QoL evolves with ageing (Beaumont and Kenealy 2004; Higgs et al. 2005).

Within nursing homes, the QoL of residents has also gained attention as an outcome for service quality. Nursing home residents generally report moderate levels of QoL (Degenholtz et al. 2006; Kane et al. 2005; King et al. 2012; Lai et al. 2015; Tu et al. 2006). Various studies have explored residents’ perceptions and experiences on what might constitute a good QoL in this setting (e.g. Bergland and Kirkevold 2006; Burack et al. 2012; Cooney et al. 2009; Kane et al. 2003; Murphy et al. 2007; Schenk et al. 2013). Not limited to health issues, QoL in nursing homes is influenced by a multitude of aspects and dimensions. However, due to the frequent physical or cognitive limitations in this phase of life and context, empirical research on QoL is often reduced to health-related QoL and/or quality of care (Courtney et al. 2006; Kane et al. 2005). These concepts are undoubtedly important but cannot replace the multidimensional conceptualisation of QoL, since they overlook relevant dimensions other than health or care (Courtney et al. 2006).

Due to the frailty status of residents (Gerritsen et al. 2004; Haugan 2014), nursing homes have a responsibility for creating an enabling environment that enhances residents’ QoL (Bergland and Kirkevold 2006; Murphy et al. 2007; Wilkinson et al. 2012). Multidimensional QoL surveys for nursing home residents exist, such as a survey developed by Kane et al. (2004), which measure residents’ wellbeing. However, they do not necessarily provide tools allowing nursing homes to take actions. As QoL measurements are a mix of personal and contextual factors, it remains vague as to how and to what extent the nursing home can provide a comprehensive context, a multidimensional set of conditions to improve their residents’ QoL.

A possible framework for nursing homes to work on their residents’ QoL is active ageing (AA). Developed by the WHO, AA is an international policy concept which encompasses *“the process of optimizing opportunities for health, participation and security in order to enhance the QoL as people age”* (WHO 2002, p. 12). Its overall aim is to provide a framework to optimise older people’s QoL, starting from their needs, wishes and expectations (WHO 2002; Walker 2002). AA acknowledges a shared responsibility for a good life in old age. It *“allows people to realise their potential for wellbeing throughout their lives and to participate in society according to their needs, desires and capabilities, while providing them with adequate protection, security and care when they need assistance”* (WHO 2002, p. 12). On the one hand, older people know best what a good QoL entails for them, since they have their own individual capabilities and attributes to enhance their QoL. Society, on the other hand, also has a role, since QoL is co-determined by structural, organisational and societal influences (Foster and Walker 2013; WHO 2002). The WHO (2002) provides a set of AA determinants that all influence QoL.

A qualitative study by Van Malderen et al. (2013) explored the potential of introducing the AA concept within the nursing home setting by hearing different stakeholders, including residents. An AA policy for nursing homes was perceived as a possible added value for the nursing home operation, since it may support them to accommodate their residents with a context that structurally enhances their QoL. Furthermore, AA is a strong international policy concept. In order to be able to implement this concept in nursing homes, the stakeholders identified nine nursing home AA determinants (Van Malderen et al. 2013). They are delineated in the next paragraph.

The *cultural* determinant refers to providing a familiar and open, welcoming atmosphere. Regarding the *behavioural* determinant, nursing homes may inform and sensitise residents about healthy lifestyles, but may not impose them and enjoyment should come first. *Psychological factors* are another determinant, including support in cognition, self-efficacy and coping. The determinant *physical environment* reflects issues of comfort, safety inside and outside the facility and privacy, as well as the goal that a personal and home-feeling should be stimulated. A nursing home should also work on the *social environment* determinant by providing social support to the residents, combatting any form of abuse, providing opportunities to learn new things and, foremost, having an open and respectful daily communication. The *economic determinant* regards fulfilling the wish of residents who want to be useful (e.g. volunteering) and guaranteeing social protection as well as transparency in costs. Due to common functional long-term needs of the residents, *care* is also identified as a determinant. The physical and emotional care should be individualised and coordinated. Another determinant *meaningful leisure*, contributes to QoL in nursing homes by offering not only a varied set of activities, but also by hearing the wishes of the residents and providing them opportunities to engage in meaningful activities. Finally, as a key issue in the AA discourse, *participation* was identified as a determinant which needs specific attention in the nursing home. The participation of residents must be seen on different levels. On the individual level, the autonomy of the resident should be supported. On the organisational level, activating residents and involving them in the policy and daily operation of the nursing home is desirable. On the societal level, the nursing home can help residents to integrate in the community.

To summarise, the AA concept can serve as a policy tool for nursing homes to assess whether they provide an environment that optimises their residents’ QoL according to their preferences and wishes (Van Malderen et al. 2013). The present study aimed at analysing the current AA status in nursing homes in Flanders. It sought to examine to what extent, according to the residents, AA policy and its various determinants are already embedded in the nursing homes and what importance residents attach to these determinants. In addition, the residents’ QoL was assessed in order to examine its relation with the residents’ AA experience in the nursing home. The hypothesis was put forward that residents having a better AA experience in the nursing home would report higher levels of QoL. A second hypothesis that was tested was that residents’ demographical features influence the AA experience and QoL, since contextual influences of QoL are also culturally and individually determined.

## Methods

### Design

A cross-sectional study was performed, using a representative sample of randomly recruited nursing home residents in Flanders, Belgium. The outcome measures were the AA operation of the nursing home as perceived by the residents (using a newly developed AA survey for nursing homes) and the QoL of residents (using the anamnestic comparative self-assessment scale (ACSA)). This study was approved by the Ethical Committee of the Universitair Ziekenhuis Brussel, VUB, Belgium (B143201215540/I/U).

### Participants

The current study was limited to nursing home residents without dementia, who account for 55 % of the Flemish nursing home population. Power calculations were made, based on a Flemish nursing home population of 70,000 residents, a confidence level set at 95 %, a margin of error set at 5 % and a standard deviation of 0.5. This led to a representative sample which was randomly recruited. First, nursing homes were stratified according to organisation type (public, private non-profit, private for-profit) and province. This was followed by a random selection of nursing homes (by means of the relevant function in SPSS). In order to have a good distribution of residents across the nursing homes, a maximum of 10 residents per facility were included. Nursing homes were asked to provide a list of all residents (without a diagnosis of dementia) living in the facility, after which the respondents were randomly selected. Those selected were invited and were included in the study if they were willing to participate. In total, 383 residents of 57 nursing homes took part. After informing the residents and obtaining their informed consent, the survey was administered face-to-face in the privacy of their rooms.

### Measurements

#### Demographic features

Gender, age, education level, previous profession, relational status and length of stay in the nursing home were noted, given their possible mediating roles in the QoL of nursing home residents, (Cooney et al. 2009; Hjaltadottir and Gustafsdottir 2007; Tseng and Wang 2001; Tu et al. 2006; Wilkinson et al. 2012). For the same reason, the functional status of the residents was assessed by means of the Katz-scale of basic activities of daily living (Katz et al. 1963).

#### AA-survey: AA operation of the nursing home

In order to examine the perceived AA operation of the nursing home, the nine AA determinants derived in the qualitative study that was described in the introduction (Van Malderen et al. 2013) were transformed into a structured questionnaire format. The operationalisation was based on extensive discussions of an expert team (gerontologists, geriatrician, psychologist, occupational therapist, and educationist) with expertise in survey-development, in working/communicating with nursing home residents, and in AA and QoL. This process resulted in an instrument composed of 61 statements, which was called the Nursing Home Active Ageing (NHAA) survey.

Participants were asked to relate to each statement. In order to enhance correct answering and avoid missing values, several criteria were taken into account (De Vaus 2002). These included the use of clear, and simple formulated statements (Wilde et al. 1994), based on the wording given by the respondents in the qualitative study, and the use of positively and negatively formulated statements to avoid unilateral answering patterns. All statements were formulated in the ‘I’-form, in order to allow the residents to identify themselves with the content. The 61 statements of the NHAA survey, covering the nine AA determinants can be found in Table 1 (translation of the Dutch NHAA survey).

**Table 1:** The 61 statements of the active ageing survey for nursing homes and the corresponding determinants

In my opinion…	
1	A lot of attention is paid to our hygiene (care)
2	The nursing home is situated in a comfortable neighbourhood (e.g. bench seats, wide sidewalks,…) (physical environment)
3	We are offered healthy food (behavior)
4	The number of excursions/trips is too small (social environment)
5	The staff works on training our memory (psychological factors)
6	Our privacy is well respected (physical environment, participation)
7	The offered activities are adjusted to my wishes, requests (meaningful leisure)
8	There is a large number of exercises (e.g. gym, sports,…) offered (behavior)
9	My room and those of others are not very spacious (physical environment)
10	Contacts between residents are limited (social environment)
11	We are adequately informed about the costs of our stay (economic factors)
12	My medical situation is well monitored (care)
13	Birthdays can be celebrated even better (meaningful leisure)
14	The staff also takes into account what I still am able to do, what my qualities are (care)
15	I am always invited to the resident council (participation)
16	The staff is not well informed about my life before my admission in the nursing home, about my life history (care, participation)
17	We are as residents well aware of the house rules, regulations of the nursing home (Social environment)
18	I do not always feel safe in our nursing home (psychological factors, care)
19	The staff regularly has a chat with us (psychological factors, care)
20	Smoking is discouraged as much as possible (behavior)
21	The staff might sometimes be distant to us (social environment)
22	The cooperation between staff members runs smoothly (care)
23	The number of activities is too small (meaningful leisure)
24	Decisions are sometimes made without my consent (participation)
25	I can choose to decorate my room according to my own taste (e.g. colours of the walls, having my own furniture in the room,…) (physical environment)
26	The drinking of alcohol is discouraged as much as possible (behavior)
27	The nursing home is open-minded towards innovations (culture)
28	I am not adequately helped/supported when I am having a hard time emotionally (psychological factors)
29	We are offered the possibility to do volunteering work/to help (economic factors, participation)
30	The care is completely tailored to me as a person (care)
31	We are encouraged each time to participate in an activity (meaningful leisure, behavior)
32	The public living spaces are decorated in a homely, cosy way (physical environment)
33	The staff treats us like children (social environment)
34	There is insufficient public or adapted transport to be able to go outside whenever I want/need (physical environment)
35	I am always offered meaningful activities whenever I wish for it (economic factors, participation)
36	I am always given the possibility to wash/clothe,…myself or go to the bathroom independently as far as it is possible to me (participation)
37	I receive a lot of affection from the staff (care)
38	Loneliness of residents is not well noticed(social environment)
39	The variety of the activities is limited (meaningful leisure)
40	Our nursing home is situated in a lively neighbourhood (e.g. centre of village/city, close to shops, …) (physical environment)
41	We are given the opportunity to discuss together with staff or other residents more sensitive topics, including palliative care, euthanasia, inheritance topics,… (psychological factors)
42	The people that work here are well acquainted with the mission/vision of the nursing home (culture)
43	I received all extra care, support and guidance I needed in the beginning of my stay to adapt more easily to my new situation (psychological factors)
44	A lot of activities are offered (meaningful leisure)
45	Only few activities are organised which can be joined by people from the neighbourhood (social environment)
46	We, as residents, can have influence in the operation of the nursing home, for example via the resident council (participation)
47	Changes in my care needs are insufficiently monitored, noticed (care)
48	We always have the possibility in this building to come together and gather with others (residents or family) (physical environment)
49	There is a familiar, convivial atmosphere in this nursing home (culture)
50	There are enough organised occasions where it is possible as resident to learn new things (e.g. The use of a computer, different languages, …) (social environment)
51	I am given responsibilities in the nursing home (participation)
52	Staff only engages in superficial conversations with us (participation)
53	People of the neighbourhood regularly come to our nursing home (Social environment)
54	As residents, we can completely choose our own life style (e.g. choosing in which activities we want to participate, if we drink alcohol, choosing what and when we eat, if we want to go outside, …) (behavior)
55	I feel valued as a person within the nursing home (participation)
56	I can maintain my hobbies from home (meaningful leisure)
57	I am encouraged to participate in activities (meaningful leisure)
58	There is an interest in my stories (psychological factors, care)
59	The neighbourhood of the nursing home is safe (physical environment)
60	Staff is respectful towards me (psychological factors, care)
61	Attention is paid to my personal hygiene (care)

Both the experienced reality and the subjective importance were considered per statement. That is, each item was evaluated by the residents on 2 Likert scales: one for the experienced reality—using a 5-point scale (ranging from totally disagree to totally agree); and one for the subjective importance of the item, using a 3-point scale (ranging from not important to very important). To weigh both scales, a NHAA score was computed according to the formula of Wilde et al. (1994). This formula generates the highest scores when experienced reality and subjective importance are rated high, and the lowest scores when experienced reality is rated low, but subjective importance is high. In addition, it ensures that higher experienced reality combined with lower subjective importance generates a higher NHAA score than lower scores on both scales. The formula is the following: subjective importance x (2× experienced reality—subjective importance). For this NHAA score, the experienced reality scale was reduced to a three point scale (merging the two disagree scores and the two agree scores) in order to obtain equivalent scales.

For the purpose of illustration, when residents experienced a low realisation of an item (Likert score 1) and they attached a lot of importance to that issue (Likert score 3), the resulting NHAA score was [3 × (2 × 1−3)] = −3. This score is lower than that when they encountered a low realisation of an item (Likert score 1) to which they did not attach any importance (Likert score 1), for which the NHAA score is [1× (2×1−1)] = 1. Higher NHAA scores refer to more positive AA experiences. The development of the NHAA survey was followed by a pilot test with 10 nursing home residents to examine the feasibility and practicality of the instrument (De Vaus 2002) and to make improvements based on the feedback of the respondents. Due to possible physical limitations (e.g. sight, hand motor skills) of the nursing home residents, this pilot study revealed the need to administer the survey in-person instead of using written self-report.

#### Quality of life

The Anamnestic Comparative Self-Assessment (ACSA) (Bernheim and Buyse 1984) was used to assess the QoL of the participant. ACSA is a biographical self-anchoring rating scale to measure the overall QoL. The anchors are the participants’ memories of their best (rating +5 on the scale) and worst (rating −5) periods in their life experience. The participants evaluate their current QoL on this individualised scale. ACSA acknowledges the individual and dynamic nature of QoL and aims to counter social desirable and peer-relative answering patterns since residents have to make comparisons with respect to their own lives (Bernheim 1999).

### Data analysis

The statistical package of SPSS (version 22.0) was used. The analysis followed a staged process. First, internal reliability was calculated for the NHAA survey using Cronbach’s alpha. Second, descriptive statistics were executed for the demographic variables and outcome measures. Third, the correlation between the two outcome measures (NHAA and QoL) was calculated and multivariate hierarchical regressions were chosen to examine the associations between the AA determinants and the QoL. Three models were used. The first model encompassed the demographic variables of the residents. In the second model, the total AA variable was further included to examine its contribution to QoL. In the third model, the nine different AA determinants replaced the total AA variable. Fourth and last, additional regression analyses were executed to examine the relation between the demographic features of the residents, their overall NHAA experience and their NHAA experience per determinant.

## Results

The AA survey emerged as a reliable instrument, with Cronbach’s alphas of 0.87 for the overall NHAA score, and, respectively, 0.91 and 0.92 for the overall experienced reality and overall subjective importance scales.

### Descriptives

Table 2 shows the main demographic features of our sample of 383 nursing home residents. The mean age was 85.6 years (SD = 6.9, min 62-max 102), and the average length of stay was 38.3 months (SD = 35.1, min 1—max 192). The majority (70.4 %) was dependent on at least two basic activities of daily living, with 17 % being independent on all basic activities of daily living.

**Table 2:** Personal characteristics of the nursing home residents

	*N*	(%)
Gender		
Male	105	27.4
Female	278	72.6
Relational status		
In a relationship	61	15.9
Not in a relationship	322	84.1
Katz-score		
O	93	29.7
A	80	25.6
B	87	27.8
C	53	17.0
Education level		
No school	2	0.5
Primary school	108	28.2
Secondary school (partially or complete)	225	58.7
High school/college	48	12.5
Previous job		
Labourer	143	37.3
White collar worker	103	26.9
Independent	63	16.4
Housewife	69	18.0
Unclear	5	1.3

Katz scores: *O* no dependencies, *A* dependencies for washing and/or clothing and for transfers and/or toilet visit, *B* additional dependencies for eating and/or having continence problems, *C* dependencies for all basic activities of daily living

Based on the NHAA score, the participants had an overall AA experience of 73.1 % in their nursing home (see Table 3) and had a positive QoL score (ACSA, $\bar{\text{x}}\,$ = 1.75 (SD = 2.25)). Table 3 shows that the AA experience differed among the AA determinants. The culture determinant had the highest score (81.9 %) while the social environment scored the lowest with 60 %. Meaningful leisure and participation did not surpass the level of 70 %. All other determinants scored between 71.2 and 80.4 %. In addition, as shown in Table 3, discrepancies between the experienced reality and subjective importance were present on all determinants, with the subjective importance attached to the determinant scoring higher than the experienced realisation of it.

**Table 3:** Descriptives on the nursing home active ageing survey and the active ageing determinants

	Experienced reality			Subjective importance			Active ageing index		
	Mean (SD)	Max	(%)	Mean (SD)	Max	(%)	Mean (SD)	Min–Max	(%)
Culture	12.0 (2.2)	15	79.7	7.73 (1.24)	9	85.9	20.49	−9 to 27	81.9
Behaviour	24.1 (3.6)	30	80.4	15.29 (2.25)	18	84.9	40.03	−18 to 54	80.6
Psychological factors	29.7 (5.4)	40	74.3	20.53 (2.96)	24	85.5	46.44	−24 to 72	73.4
Physical environment	35.02 (4.96)	45	77.8	22.92 (3.04)	27	84.9	57.23	−27 to 81	78.0
Social environment	29.08 (5.19)	45	64.6	20.36 (3.51)	27	75.4	38.78	−27 to 81	60.9
Economic factors	10.49 (2.55)	15	69.9	6.65 (1.70)	9	73.9	16.38	−9 to 27	70.5
Care	51.74 (7.49)	65	79.6	35.00 (3,29)	39	89.7	88.42	−39 to 117	81.7
Meaningful leisure	32.43 (6.02)	45	72.1	20.99 (5.08)	27	77.7	48.16	−27 to 81	69.6
Participation	34.51 (5.42)	50	69.0	23.00 (3.52)	30	76.7	52.97	−30 to 90	69.1
Overall Active Ageing	225.23 (28.47)	305	74.0	149.91 (18.00)	183	81.9	351.90	−183 to 549	73.1

### Multivariate analysis

Based on the bivariate analysis, a significant positive correlation was found between the overall AA index and the ACSA (*r* = 0.378, *p* < 0.05), implying that a higher AA experience of the nursing home operation accords with a higher perceived QoL.

The results of the multivariate analyses with the residents’ QoL as the dependent variable can be found in Table 4. Model 1 shows no significant relations between the demographic and functional variables and QoL. When controlling for demographic variables, model 2 reveals that the total AA operation of the nursing home was positively related to the QoL of the residents (Table 4). These results correspond with the hypothesis on positive relationships between an experienced AA nursing home context and residents’ QoL. Model 3 demonstrates that, after controlling for the demographic variables, two AA determinants were significantly positively associated with the residents’ QoL: psychological factors and participation.

**Table 4:** Multivariate analyses of background and active ageing variables and the quality of life among nursing home residents

	Model 1	Model 2	Model 3
	Beta	Beta	Beta
Gender	−0.078	−0.103*	−0.103
Age	0.032	0.035	0.035
Education	−0.107	−0.078	−0.078
Relational status	−0.008	−0.023	−0.023
Length of stay in nursing home	0.039	−0.068	−0.068
BADL-dependency	−0.081	−0.088	−0.088
TOTAL active ageing operation		0.412*	
Active ageing determinant: CULTURE			0.059
Active ageing determinant: BEHAVIOUR			0.042
Active ageing determinant: PSYCHOLOGICAL FACTORS			0.227*
Active ageing determinant: PHYSICAL ENVIRONMENT			−0.063
Active ageing determinant: SOCIAL ENVIRONMENT			0.107
Active ageing determinant: ECONOMICAL FACTORS			0.002
Active ageing determinant: CARE			−0.052
Active ageing determinant: MEANINGFUL LEISURE			0.068
Active ageing determinant: PARTICIPATION			0.197*
	*R* ^2^	*R* ^2^	*R* ^2^
	0.024	0.186*	0.229*

* *p* < 0.05

With respect to our last hypothesis on the relation between the demographic variables and the NHAA scores, the results of the corresponding analyses can be found in Table 5. The education level of the residents appeared to be the best demographic predictor for the NHAA experience with significant negative relationships with the overall NHAA score, as well as with the various AA determinants, except for the economic determinant. Other significant results showed a positive relationship between age and the care determinant, a positive relationship between residents’ relational status and meaningful leisure and a negative relation between dependency on basic activities of daily living and the participation determinant.

**Table 5:** Multivariate analyses of background variables and Active Ageing experience among nursing home residents

NHAA										
	Culture	Behaviour	Psychological Factors	Physical environment	Social environment	Economical factors	Care	Meaningful leisure	Participation	Total
	Beta									
Gender	−0.005	0.069	−0.022	0.001	0.069	−0.065	−0.067	0.116	−0.019	0.017
Age	0.050	0.038	0.058	0.107	0.095	−0.107	0.132*	−0.020	−0.006	0.087
Education	−0.144*	−0.178*	−0.182*	−0.131*	−0.188*	−0.099	−0.188*	−0.145*	−0.152*	−0.215*
Relational status	−0.040	0.078	−0.058	−0.022	−0.008	0.058	−0.040	0.127*	0.028	−0.008
Length of stay	0.039	0.017	−0.058	−0.012	−0.016	0.071	−0.057	0.062	0.031	−0.039
BADL-dependency	−0.005	0.029	0.010	−0.004	0.030	−0.014	0.014	0.042	−0.155*	−0.020
	*R* ^2^									
	0.028	0.049*	0.040	0.029	0.057*	0.032	0.054*	0.062*	0.048*	0.054*

* *p* < 0.05

## Discussion

Our study examined the extent to which nursing homes apply an AA-oriented approach, based on the experiences of the residents, to the residents’ QoL. By measuring the experienced presence of a multidimensional set of environmental factors that may optimise a resident’s QoL, the study builds further on earlier multidimensional QoL studies in nursing homes (e.g. Burack et al. 2012; Degenholtz et al. 2006; Kane et al. 2004; Murphy et al. 2007; Schenk et al. 2013). The new NHAA survey, which was developed for the current research, appears to be a reliable instrument, and was able to demonstrate a weak positive correlation with QoL. This result indicates that the more residents experience an AA-oriented approach in the nursing home, the higher their QoL. Although the link between AA and QoL has been extensively described, it often does so theoretically without empirically establishing the relationship between the two. To our knowledge, this association has not been previously considered in the nursing home context.

Overall, based on the descriptive analyses, the residents were found to be rather positive regarding the AA operation of their nursing home, and had a moderately positive evaluation of their QoL. The positive QoL that was reported here corresponds with studies elsewhere (Degenholtz et al. 2006; Kane et al. 2004; King et al. 2012; Lai et al. 2015; Tu et al. 2006) and counters the widely held societal prejudice that people in nursing homes are unhappy and experience a poor QoL. This might partly be explained by the dynamic characteristic of QoL (Bowling and Gabriel 2004) in which people change their meaning of the phenomenon. As the Selection–Optimisation–Compensation model for successful ageing of Baltes (1997) indicates, people select their goals (selection) within the context they live in; they choose methods to achieve these goals (optimisation) and alter them if the methods they previously preferred are no longer possible (compensation). Also nursing home residents reconstruct their conceptions of a good life when living in a nursing home (Bergland and Kirkevold 2006) and develop cognitive strategies to cope with new life situations (Custers et al. 2013).

Looking at the respective AA determinants in detail, residents had the most positive AA experience with two particular determinants: culture and care. The high score for the care determinant are in line with other studies examining residents’ experiences on the care provided showing positive results (Nakrem et al. 2011). The residents were also relatively satisfied with the psychological aspects, the behavioural domain, the physical environment and the economic factors, resulting in NHAA-scores ranging between 72 and 80 %. The results for the psychological factors might be seen as a positive surprise, since in Flanders, nursing homes are not legally bound to employ personnel with a psychological background (Agency Care and Health 2015), and care providers do not always feel that they have the expertise to provide adequate psychosocial care (Isola et al. 2008). A systematic review by Bradshaw et al. (2012) supports our results on the physical environment, showing the importance of the presence of homelike environments in nursing homes.

The lowest AA experience was encountered for the social environment domain (60 %). Our results underline the need in nursing homes for further efforts in social support, in order to enhance and maintain close, personal relationships. The need for social support is also revealed by other studies, showing that residents find it difficult to keep contact with former friends (Boelsma et al. 2014) and to establish new contacts with residents (Drageset et al. 2011). They also frequently feel neglected and ignored when trying to bond with staff (Nakrem et al. 2011).

Our study also shows that the AA domains of meaningful leisure and participation are not realised to the fullest (NHAA scores <70 %). Nursing homes need to provide a variety of stimulation and activities that give residents the feeling of belonging (Schenk et al. 2013) and of meaningfulness. Not all activities are, however, necessarily perceived as meaningful by the residents (Bergland and Kirkevold 2006). Nursing homes should invest in more and substantive leisure time (Harper-Ice 2002; den Ouden et al. 2015), building on residents’ wishes and competences. With respect to the participation determinant, other studies have shown that residents have only limited choices or opportunities to be heard and are often poorly informed (Abbott et al. 2000). Suggestions of residents are frequently seen by the staff as trivial and are easily ignored, in order to avoid disruptions of daily routines (Harnett 2010).

The multivariate analyses on QoL confirm our initial hypothesis. Significant associations between the nursing homes’ overall AA operation modus and the residents’ QoL were observed, after controlling for the demographic and functional characteristics of the residents. This emphasises the importance of comprehensive, multidimensional strategies in nursing homes to enhance residents’ QoL. However, the AA context only explains some 20 % of the QoL variance, suggesting that there are other important factors contributing to an optimal QoL in the nursing home. These factors might be the personality and mental attitude of the residents (Bergland and Kirkevold 2006; Cooney et al. 2009), their family situation, life experiences and life events, health status, expectations and adaptive responses (Cooney et al. 2009).

The results also showed significant (positive) relationships between the determinants psychological factors and participation and QoL. The added values of both of these determinants for residents’ QoL are supported by psychological and participation intervention studies in nursing homes, which show positive effects on the residents’ QoL (e.g. Chang et al. 2008; Cook 1998; Haight et al. 2000; Knight et al. 2010; Lee et al. 2009; Yuen et al. 2008).

With respect to our hypothesis that demographic features influence the AA experience, the multivariate analyses show that the residents’ educational level has the highest predictive value on their NHAA experience. Moreover, higher educational attainment was negatively associated with most of the AA determinants and with the overall AA experience. It is known that highly educated people might be more critical about their situation and institutions and less willing to accept circumstances (Cools et al. 2010). Furthermore, at this point of time, highly educated people are still a minority among the oldest old and in nursing homes, leading to a nursing home operation not necessarily adapted to the needs and wishes of their highly educated residents. The educational level of the new generations of older people is increasing, however, with more than half of them graduating from the University College (Tepper and Cassidy 2004). This trend may initiate new challenges for the nursing home, which have to adapt to this new group of older people. The nursing home will have to provide an optimal AA functioning for a large variety of older people.

In addition to the educational level, age was positively related to the AA care experience. The question arises if older people are in fact more pleased or less eager to complain about their care than younger residents. Chou et al. (2003) postulate that older residents might become more easy-going and accepting. Also, the relational status of the residents was positively related with their AA meaningful leisure experience, as residents who were still in a relationship were more fulfilled with the organisation of their leisure time by the nursing home. A study by Janke et al. (2008) revealed that older people who are still married are less in need of being involved in organised activities for their wellbeing in comparison to widowed people. Finally, dependency for basic activities of daily living status was negatively correlated with the AA participation experience. This might imply that nursing homes do not provide the same opportunities to participate for residents who have more functional long-term care needs. Corresponding results can be found in a study by Hwang et al. (2006). Also, functional limitations might prohibit the residents’ ability to properly articulate their wishes. This result might reflect the existing tension in nursing homes between safety and choice and freedom (Kane and Kane 2001). Due to their more vulnerable position, safety issues might be prioritised by nursing homes, at the expense of the residents’ participation (Kane and Kane 2001). Since, however, only a minority of the residents preferred being safe over being free (Degenholtz et al. 1997), nursing homes should provide an enabling and empowering context for residents, despite their possible functional long-term needs.

A few limitations of the present study have to be taken into account. Despite efforts made to ensure confidentiality and anonymity for our respondents, socially desirable answers can never be completely ruled out. Power imbalances between staff and residents might frighten residents to speak freely. Residents want to avoid causing trouble and might lower their expectations (Nakrem et al. 2011). Another limitation is that we focused on residents without dementia, since those with cognitive impairment might need a different approach. Therefore, a large part of the resident population (estimated at 45 %) was excluded. Still, further research could be performed on examining the AA satisfaction and QoL of people with dementia living in nursing home facilities. Furthermore, we only focused on the Dutch-speaking region of Belgium. Our results are consequently not necessarily generalizable to the whole country or to other countries.

However, this study also has several strengths. The NHAA survey encompasses the determinants experienced as relevant for residents’ QoL. The statements in the NHAA survey were developed based on focus groups with stakeholders and residents themselves. Furthermore, in line with the AA premises, this study examined and weighed per participant the importance attached to each of the items, since only the residents themselves can determine what is important for them. We also provide a comprehensive overview of the AA approach of nursing homes, based on what is important to residents, in relation to the residents’ QoL. This is based on a rather large and representative, randomly recruited sample of nursing home residents.

In sum, our study integrated the AA concept of the WHO into the setting of the nursing home and assessed the nursing home’s functioning in relation to AA, including the residents’ ratings of importance. AA underscores the need for a holistic vision and a multidisciplinary approach to optimise residents’ QoL, starting from residents’ competences, wishes, and participation. A nursing home approach that provides a comprehensive AA context might help to contribute to the residents QoL.

The measurements used in this study are not intended to point the finger at nursing homes but serve as an incentive for quality improvement (considering trends over time) with a long-term commitment. Based on our results, we conclude that nursing home residents in Flanders (Belgium) have a relative positive experience regarding the AA approach of their nursing home and in general have a positive QoL. Still, further AA efforts are needed, mainly with respect to participation, meaningful leisure and the social environment.

Looking to the future, nursing homes have to adapt to a new generation of older people, who are more highly educated and have specific standards, wishes and needs. Presently, the needs of highly educated people appear to be overlooked, resulting in a lower AA experience. The future nursing home will include an unprecedented heterogeneity of older people. Now is the time to prepare for these changes, in order to keep the QoL of the residents high.

Most importantly, due to the increasing heterogeneity, more individualised programs will be helpful. It is also important to anticipate as nursing home policy and operation on the next nursing home population of articulate baby boomers. This can be done by focusing on more opportunities to remain in control, active and to participate, better contacts between like-minded residents, a larger variety of meaningful leisure, adapted and differentiated communication towards the different residents.

Notwithstanding that the AA concept is less known in their daily practice, nursing homes are already AA-minded in their mode of operation, but further work and AA realisations are possible and necessary. Since the NHAA survey developed for this study starts from the opinions and wishes of the residents, this survey might be eligible to be implemented in nursing homes as (for example, yearly) quality measure in order to monitor and optimise their quality on each of the different AA determinants which will help nursing homes for future challenges.

## References

[CR1] Abbott S, Fisk M, Forward L (2000). Social and democratic participation in residential settings for older people: realities and aspirations. Ageing Soc.

[CR2] Agency Health and Care (Agentschap Zorg en Gezondheid, Departement Welzijn, Volksgezondheid en Gezin, Zorginspectie) (2015) Interpretatie van de Erkennings-voorwaarden en –normen voor woonzorgcentra (wzc) rust- en verzorgingstehuizen (rvt) en centra voor kortverblijf (cvk) ingebed in een woonzorgcentrum. Agentschap Zorg en Gezondheid, Brussels

[CR3] Baltes PB (1997). On the incomplete architecture of human ontogeny: selection, optimization and compensation as foundation of developmental theory. Am Psychol.

[CR4] Beaumont JG, Kenealy PM (2004). Quality of life perceptions and social comparisons in healthy old age. Ageing Soc.

[CR5] Bergland A, Kirkevold M (2006). Thriving in nursing homes in Norway: contributing aspects described by residents. Int J Nurs Stud.

[CR6] Bernheim J (1999). How to get serious answers to the serious question: ‘how have you been’: subjective QoL as an individual experiential emergent construct?. Bioethics.

[CR7] Bernheim JL, Buyse M (1984). The anamnestic comparative self-assessment for measuring the subjective quality of life for cancer patients. J Psychosoc Oncol.

[CR71] Boelsma F, Baur VE, Woelders S, Abma  TA (2014). “Small” things matter: residents’ involvement in practice improvements in long-term care facilities. J Aging Stud.

[CR8] Bowling A, Gabriel Z (2004). An integrational model of QoL in older age, results from QoL survey in Britain. Soc Indic Res.

[CR9] Bowling A, Gabriel Z, Dykes J, Marriott Dowding L, Evans O, Fleissig A, Banister D, Sutton S (2003). Let’s ask them: a national survey of definitions of quality of life and its enhancement among people aged 65 and over. Int J Aging Hum Dev.

[CR10] Bradshaw SA, Playford ED, Riazi A (2012). Living well in care homes: a systematic review of qualitative studies. Age Ageing.

[CR11] Burack OR, Weiner AS, Reinhardt JP, Annunziato RA (2012). What matters most to nursing home elders: quality of life in the nursing home. JAMDA.

[CR12] Chang S, Wung S, Crogan NL (2008). Improving activities of daily living for nursing home elder persons in Taiwan. Nurs Res.

[CR13] Chou S, Boldy DP, Lee AH (2003). Factors influencing residents’ satisfaction in residential aged care. Gerontologist.

[CR14] Cook EA (1998). Effects of reminiscence on life satisfaction of elderly female nursing home residents. Health Care Women.

[CR15] Cools M, De Ruyver B, Easton M (2010). Safety, societal problems and citizens’ perceptions: new empirical data, theories and analyses.

[CR16] Cooney A, Murphy K, O’Shea E (2009). Resident perspectives of the determinants of quality of life in residential care in Ireland. J Adv Nurs.

[CR17] Courtney M, Edwards H, Stephan J, O’Reily M, Duggan C (2006). Quality of life measures of aged care facilities: a literature review. Australas J Ageing.

[CR18] Custers AFJ, Westerhof GJ, Kuin Y, Gerritsen DL, Riksen-Walraven JM (2013). Need fulfillment in the nursing home: resident and observer perspectives in relation to resident well-being. Eur J Ageing.

[CR19] De Vaus D (2002). Social surveys.

[CR20] Degenholtz H, Kane RA, Kivnick HQ (1997). Care-related preferences and values of elderly community-based LTC consumers: can case managers learn what’s important to clients?. Gerontologist.

[CR21] Degenholtz HB, Kane RA, Kane RL, Bershadsky B (2006). Predicting nursing facility residents’ quality of life using external indicators. Health Serv Res.

[CR72] den Ouden M, Bleijlevens MHC, Meijers JMM, Zwakhalen SMG, Braun SM, Tan FES, Hamers JPH (2015). Daily (in)activities of nursing home residents in their wards: an observation study. J Am Med Dir Assoc.

[CR73] Drageset J, Kirkevold M, Espehaug B (2011). Loneliness and social support among nursing home residents without cognitive impairment: a questionnaire survey. Int J Nurs Stud.

[CR22] Eurostat (2012) Active Ageing and solidarity between generations. A statistical portrait of the European Union 2012. European Union, Belgium

[CR23] Foster L, Walker A (2013). Gender and active ageing in Europe. Eur J Ageing.

[CR24] Gerritsen DI, Steverink N, Ooms ME, Ribbe MW (2004). Finding a useful conceptual basis for enhancing the quality of life of nursing home residents. Qual Life Res.

[CR25] Gilhooly M, Gilhooly K, Bowling A, Walker A (2005). Quality of life, meaning and measurement. Understanding quality of life in old age.

[CR26] Haight BK, Michel Y, Hendrix S (2000). The extended effects of life review in nursing home residents. Int J Aging Hum.

[CR27] Harnett T (2010). Seeking exemptions from nursing home routines: residents’ everyday influence attempts and institutional order. J Aging Stud.

[CR74] Harper-Ice G (2002). Daily life in a nursing home. Has it changed in 25 years. J Aging Stud.

[CR28] Haugan G (2014). Meaning-in-life in nursing-home patients: a valuable approach for enhancing psychological and physical well-being?. J Clin Nurs.

[CR29] Higgs P, Hyde M, Arber S, Blane D, Breeze E, Nazroo J, Wiggins D, Walker A (2005). Dimensions of the inequalities in quality of life in older age. Understanding quality of life in old age.

[CR30] Hjaltadottir I, Gustafsdottir M (2007). Quality of life in nursing homes: perceptions of physically frail elderly patients. Scand J Caring Sci.

[CR75] Huber M, Rodrigues R, Hoffmann F, Gasior K, Marin B (2009). Facts and figures on long-term care. Europe and North America.

[CR31] Hwang H, Lin H, Tung Y, Wu H (2006). Correlates of perceived autonomy among elders in a senior citizen home: a cross-sectional survey. Int J Nurs Stud.

[CR32] Isola A, Backman K, Voutilainen P, Rautsiala R (2008). Quality of institutional care of older people as evaluated by nursing staff. J Clin Nurs.

[CR33] Janke MC, Nimrod G, Kleiber DA (2008). Leisure activity and depressive symptoms of widowed and married women in later life. J Leis Res.

[CR34] Kane RA (2001). Long-term care and a good quality of life: bringing them closer together. Gerontologist.

[CR35] Kane RL, Kane RA (2001). What older people want from long-term care and how they can get it. Health Aff.

[CR36] Kane RA, Kling KC, Bershadsky B, Kane RL (2003). Quality of life measures for nursing home residents. J Gerontol A.

[CR37] Kane RL, Bershadsky B, Kane RA, Degenholtz HH (2004). Using resident reports of quality of life to distinguish among nursing homes. Gerontologist.

[CR38] Kane RL, Rockwood T, Hyer K, Desjardins K, Brassard A, Gessert C, Kane R (2005). Rating the importance of nursing home residents’ quality of life. JAGS.

[CR39] Katz S, Ford AB, Moskowitz RW, Jackson BA, Jaffe MW (1963). Studies of illness in the aged: the index of ADL: a standardized measure of biological and psychosocial function. JAMA.

[CR40] Kelley-Gillespie N (2009). An integrated conceptual model of quality of life for older adults based on a synthesis of the literature. Appl Res Qual Life.

[CR41] King J, Yourman L, Ahalt C, Eng C, Knight SJ, Pérez-Stable EJ, Smith AK (2012). Quality of life in late-life disability: I don’t feel bitter because I am in a wheelchair. JAGS.

[CR42] Knight C, Haslam SA, Haslam C (2010). In home or at home? How collective decision making in a new care facility enhances social interaction and wellbeing amongst older adults. Ag Soc.

[CR43] Lai CKY, Leung DDM, Kwong EWY, Lee RLP (2015). Factors associated with the quality of life of nursing home residents in Hong Kong. Int Nurs Rev.

[CR44] Lee LY, Lee DT, Woo J (2009). Tai chi and health-related quality of life in nursing home residents. J Nurs Scholarship.

[CR45] Marinelli RD, Plummer OK (1999). Healthy aging: beyond exercise. Act Adapt Aging.

[CR46] Murphy K, Shea EO, Cooney A (2007). Quality of life for older people living in long-stay settings in Ireland. J Clin Nurs.

[CR76] Murphy K, O’Shea E, Cooney A (2008). Nurse managers’ perceptions of quality of life of older adult’s living in long-stay care in Ireland. Is it time for a bill of rights?. J Gerontol Nurs.

[CR47] Nakrem S, GuttormsenVinsnes A, Seim A (2011). Residents’ experiences of interpersonal factors in nursing home care: a qualitative study. Int J Nurs Stud.

[CR48] Research Department Flemish Government (Studiedienst Vlaamse Regering) (2014) Vlaanderen in Cijfers. Studiedienst Vlaamse Regering, Brussels

[CR49] Schenk L, Meyer R, Behr A, Kuhlmey A, Holzhausen M (2013). Quality of life in nursing homes: results of a qualitative resident survey. Qual Life Res.

[CR50] Tepper LM, Cassidy TM (2004). Multidisciplinary perspectives on aging.

[CR51] Tseng S, Wang R (2001). Quality of life and related factors among elderly nursing home residents in southern Taiwan. Public Health Nurs.

[CR52] Tu Y, Wang R, Yeh S (2006). Relationship between perceived empowerment care and quality of life among elderly residents within nursing homes in Taiwan: a questionnaire survey. Int J Nurs Stud.

[CR53] Van Malderen L, Mets T, De Vriendt P, Gorus E (2013). The Active Ageing-concept translated to the residential long-term care. Qual Life Res.

[CR54] Walker A (2002). A strategy for active ageing. Int Soc Secur Rev.

[CR55] Walker A (2008). Commentary: the emergence and application of active aging in Europe. J Aging Soc Policy.

[CR56] WHOQOL Group (1995) The World Health Organization Quality of Life assessment (WHOQOL): position paper from the World Health Organization. Soc Sc Med 41:1403–1409 10.1016/0277-9536(95)00112-k 8560308

[CR57] Wilde B, Larsson G, Larsson M, Starrin B (1994). Quality of care—development of a patient-centered questionnaire based on a grounded theory model. Scand J Caring Sci.

[CR58] Wilkinson TJ, Kiata LJ, Peri K, Robinson M, Kerse NM (2012). Quality of life for older people in residential care is related to connectedness, willingness to enter care, and co-residents. Australas J Ageing.

[CR59] World Health Organization (2002). Active ageing: a policy framework.

[CR60] Yuen HK, Huang P, Burik JK, Smith TG (2008). Impact of participating in volunteer activities for residents living in long-term-care facilities. Am J Occup Ther.

